# Gut microbe-derived metabolites and the risk of cardiovascular disease in the METSIM cohort

**DOI:** 10.3389/fmicb.2024.1411328

**Published:** 2024-08-01

**Authors:** Sahereh Mirzaei, Holli A. DeVon, Rita M. Cantor, Arjen Cupido, Lilian Fernandes Silva, Markku Laakso, Aldons J. Lusis

**Affiliations:** ^1^Department of Medicine, Division of Cardiology, David Geffen School of Medicine, University of California, Los Angeles, Los Angeles, CA, United States; ^2^School of Nursing, University of California, Los Angeles, Los Angeles, CA, United States; ^3^Department of Human Genetics, David Geffen School of Medicine, University of California, Los Angeles, Los Angeles, CA, United States; ^4^Department of Vascular Medicine, Amsterdam University Medical Centers, University of Amsterdam, Amsterdam Cardiovascular Sciences, Amsterdam, Netherlands; ^5^Department of Clinical Medicine, Internal Medicine, University of Eastern Finland, Kuopio, Finland; ^6^Department of Human Genetics and Microbiology, Immunology and Molecular Genetics, David Geffen School of Medicine, University of California, Los Angeles, Los Angeles, CA, United States

**Keywords:** gut metabolites, succinate, ursodeoxycholic acid, cardiovascular disease, myocardial infarction, stroke

## Abstract

**Background:**

An association between gut microbes and cardiovascular disease (CVD) has been established, but the underlying mechanisms remain largely unknown.

**Methods:**

We conducted a secondary analysis of the cross-sectional data obtained from the Metabolic Syndrome in Men (METSIM) population-based cohort of 10,194 Finnish men (age = 57.65 ± 7.12 years). We tested the levels of circulating gut microbe-derived metabolites as predictors of CVD, ischemic cerebrovascular accident (CVA), and myocardial infarction (MI). The Kaplan–Meier method was used to estimate the time from the participants' first outpatient clinic visit to the occurrence of adverse outcomes. The associations between metabolite levels and the outcomes were assessed using Cox proportional hazard models.

**Results:**

During a median follow-up period of 200 months, 979 participants experienced CVD, 397 experienced CVA, and 548 experienced MI. After adjusting for traditional risk factors and correcting for multiple comparisons, higher plasma levels of succinate [quartile 4 vs. quartile 1; adjusted hazard ratio, aHR = 1.30, (confidence interval (CI), 1.10–1.53) *p* = 0.0003, adjusted *p* = 0.01] were significantly associated with the risk of CVD. High plasma levels of ursodeoxycholic acid (UDCA) (quartile 3 vs. quartile 1); [aHR = 1.68, (CI, 1.26–2.2); *p* = 0.0003, adj. *p* = 0.01] were associated with a higher risk of CVA. Furthermore, as a continuous variable, succinate was associated with a 10% decrease in the risk of CVD [aHR = 0.9; (CI, 0.84–0.97); *p* = 0.008] and a 15% decrease in the risk of MI [aHR = 0.85, (CI, 0.77–0.93); *p* = 0.0007].

**Conclusion:**

Gut microbe-derived metabolites, succinate, and ursodeoxycholic acid were associated with CVD, MI, and CVA, respectively. Regulating the gut microbes may represent a potential therapeutic target for modulating CVD and CVA.

## 1 Introduction

Despite advances in treatment, cardiovascular disease (CVD) remains the leading cause of death globally, contributing to morbidity and high healthcare system costs (Benjamin et al., 2019; Roth et al., 2020). CVD is influenced by genetic, environmental, and known traditional risk factors, which further complicate the understanding of the disease (Bjorkegren and Lusis, 2022). Broadly known traditional risk factors, such as high cholesterol, diabetes, obesity, and smoking, only account for a fraction of the incidence of CVD (Benjamin et al., 2019). In recent years, studies have identified gut dysbiosis and gut microbe-derived metabolites as novel risk factors for atherosclerosis, cerebrovascular disease, hypertension, and myocardial dysfunction (Drouin et al., 2019). The gut microbiome composition is highly dynamic and is an important mediator between the host's internal and external environments. It can be altered throughout the host's lifespan by various factors such as the host's genetics, diet, and medications (Spanogiannopoulos et al., 2016; Bubier et al., 2021; Rehner et al., 2023). The adaptive and malleable nature of the gut microbiome serves to optimize the host's metabolic and immune performance in response to physiological and environmental alterations, thereby maintaining physiological homeostasis and health status (Candela et al., 2014). Imbalances in the gut microbial composition and the plasma concentration of gut microbe-derived metabolites can induce metabolic shifts that result in cardiometabolic diseases including CVD (Brown and Hazen, 2015; Witkowski et al., 2020). Gut microbes produce numerous bioactive metabolites that can be absorbed into enterohepatic circulation and transported to various tissues, affecting the host's physiology (Fan and Pedersen, 2021). For example, the gut microbe-derived metabolite trimethylamine N-oxide (TMAO) has been identified as a novel risk marker and plays an important role in the onset and development of CVD and all-cause mortality. The concentration of TMAO could be used to predict the risk of incident CVD (Brown and Hazen, 2015). In the present study, the association between 35 gut microbe-derived metabolites and the risk of CVD was assessed in the population-based cohort of Metabolic Syndrome in Men (METSIM) during a 200-month follow-up. We also examined the association of these 35 metabolites with cerebrovascular accident (CVA) and myocardial infarction (MI) individually.

## 2 Materials and methods

### 2.1 Study design and sample population

In this study, we conducted a secondary analysis of the data from the METSIM population-based cross-sectional and longitudinal cohort. The METSIM cohort consisted of 10,194 men, aged between 45 and 74 years, selected from the population register of Kuopio town, Eastern Finland. The data were collected from 2005 to 2010. The prevalence and incidence of CVD and type 2 diabetes (T2D) are higher in Finnish men than in Finnish women; therefore, only men were included in the METSIM study. In addition, the sample was limited to men to increase its homogeneity. The homogenous nature of the cohort made it particularly useful for understanding host–microbiome relationships. The cohort underwent detailed phenotyping, particularly for cardiovascular- and diabetes-related traits, and had a long follow-up period of 200 months. In our study, CVD was defined as the occurrence of incident total myocardial infarction events, coronary artery disease death, and cerebral infarction events during the follow-up period. The METSIM study has been previously described in detail (Laakso et al., 2017).

### 2.2 Setting, data collection, and metabolites

During their 1-day outpatient visit at the University of Kuopio, the information regarding the patients' clinical data, health status, and medical treatments were collected. Measurements of height, weight, waist-to-hip ratio, blood pressure (BP), and fat percentages were recorded. In the clinic, fasting blood samples were collected and stored at −80°C. Laboratory analyses of fasting glucose, hemoglobin A1c (HbA1c), lipids, and high-sensitivity C-reactive protein (CRP) were performed after 12 h and have been mentioned previously (Stancakova et al., 2009). Metabolites were measured at baseline, using Metabolon's untargeted Discovery HD4 platform (Metabolon, Durham, NC, USA) with ultra-high-performance liquid chromatography–tandem mass spectroscopy. The methods have been previously described in detail (Vangipurapu et al., 2019; Yin et al., 2022). A total of 35 unique gut microbe-derived metabolites were included in the statistical analyses. We selected 35 gut microbe-derived metabolites based on their availability in the METSIM cohort. We excluded gut-derived metabolites with more than 20% missing values. All laboratory methods, such as metabolomics analysis, were performed in accordance with the relevant guidelines and regulations. The data of the METSIM participants were linked to several Finnish registries, including a hospital discharge registry, a drug reimbursement and prescription registry, and cancer and mortality registries.

### 2.3 Statistical analysis

All metabolites were log-transformed to correct for skewness. Student's *t*-test or Welch's *t*-test was used for continuous variables, and the χ^2^ test was used for categorical variables to determine significant differences between the groups. Missing values were imputed. The Kaplan–Meier survival curves were estimated and generated as right-censored with the endpoint of the diagnosis of CVD, cerebrovascular accident (CVA), and myocardial infarction (MI). The Cox proportional hazards analysis was conducted to determine the hazard ratio (HR) and 95% confidence intervals (95% CIs) for CVD, CVA, and MI stratified according to the metabolites in quartiles [quartile 4 (Q4) vs. quartile 1 (Q1)]. Adjustments were made for age, body mass index (BMI), smoking status, systolic blood pressure (SBP), low-density lipoprotein cholesterol (LDL-C), high-density lipoprotein cholesterol (HDL-C), HbA1c, triglycerides, C-reactive protein (CRP), and the estimated glomerular filtration rate (eGFR). The *p*-values were obtained and further corrected for multiple comparisons using the Bonferroni correction. The overall event-free survival was defined as the interval from the initial recruitment date to the date of follow-up when outcomes were diagnosed. The statistical analysis and visualization were performed using R Studio with R version 4.0.5.

## 3 Results

A total of 979 (11.4%) participants experienced CVD, 397 (4%) experienced CVA, and 548 (6%) experienced MI events during the follow-up period. The clinical and laboratory characteristics of the participants are shown in Tables 1, 2.

**Table 1:** Comparison of the baseline characteristics of the participants without CVD, with CVD, without CVA, and with CVA.

**Clinical traits**	**Non-CVD**	**CVD**	***p*-value**	**Non-CVA**	**CVA**	***p*-value**
Age (years) mean ± SD	57.2 (7)	61.72 (6.7)	2.3E-68	57.3 (7.05)	61.8 (6.6)	8.3E-33
BMI (Kg/m^2^)	26.5 (4.7)	27.5 (5.5)	1.7E-10	26.6 (4.2)	27.2 (4.5)	0.007
Waist-to-hip ratio	0.97 (0.08)	0.99 (0.09)	1.4E-25	0.97 (0.8)	0.99 (0.8)	2.2E-08
Fat mass (%)	23 (8.5)	26.5 (10.3)	1.6E-46	23.1 (8)	26.5 (10.5)	2.2E-16
Plasma glucose (mmol/L)	5.8 (0.8)	5.9 (0.9)	7.3E-11	5.8 (1.9)	5.8 (1.4)	0.002
Plasma insulin (mU/L)	6.8 (6.5)	8.3 (8.5)	5.1E-18	6.9 (6.7)	7.9 (7.5)	0.0002
HbA1C (%)	5.7 (0.4)	5.9 (0.6)	5.5E-43	5.7 (0.5)	5.9 (0.6)	8.0E-15
HOMA-IR	1.7 (1.8)	2.2 (2.5)	4.1E-20	1.8 (1.9)	2.1 (2.3)	4.8E-05
Total cholesterol (mmol/L)	5.2 (1.3)	5.1 (1.5)	0.01	5.2 (1.3)	5.0 (1.3)	0.008
LDL cholesterol (mmol/L)	3.2 (1.2)	3.5 (1.3)	0.025	3.2 (1.2)	3.1 (1.2)	0.01
HDL cholesterol (mmol/L)	1.5 (0.5)	1.3 (0.5)	1.6E-11	1.3 (0.5)	1.0 (0.5)	0.034
Total triglyceride (mmol/L)	1.2 (0.8)	1.3 (0.9)	5.0E-10	1.2 (0.8)	1.3 (0.8)	0.10
Systolic BP (mmHg)	136 (21.3)	142 (23.5)	2.2E-16	135 (21.3)	142 (23.3)	1.3E-10
Diastolic BP (mmHg)	87.3 (12.6)	87.3 (13.3)	0.43	87.3 (12.6)	87 (12.6)	0.90
GFR	0.09 (0.01)	0.08 (0.02)	1.2E-11	0.88 (0.01)	0.85 (0.02)	1.2E-04
CRP (mg/L)	1.06 (1.8)	1.5 (2.7)	1.6E-64	1.0 (1.9)	1.4 (2.6)	3.1E-06
Alcohol/week (gram)	62 (137)	49 (129)	1.6E-05	61 (136)	62 (138)	0.16
**Smoking [*****n*** **(%)]**						
No	3,626 (42)	343 (35)	1.0E-06	4,005 (41)	145 (36)	1.2E-05
Yes	1,517 (17.6)	230 (23.5)		1,762 (18)	81 (20)	
Ex-smoker	3,495 (40.5)	406 (41.5)		4,026 (41)	178 (44)	
**Statins [*****n*** **(%)]**						
No	6,562 (76)	679 (69.4)	6.6E-06	7,089 (72)	261 (65)	7.7E-04
Yes	2,076 (24)	300 (30.6)		2,704 (28)	143 (35)	

CVD, cardiovascular disease; CVA, cerebrovascular accident; SD, standard deviation; BMI, body mass index; HbA1c, hemoglobin A1c; HOMA-IR, homeostatic model assessment of insulin resistance; LDL, low-density lipoprotein; HDL, high-density lipoprotein; BP, blood pressure; GFR, glomerular filtration rate; CRP, C-reactive protein.

**Table 2:** Comparison of the baseline characteristics of the participants without MI and with MI.

**Clinical traits**	**Non-MI**	**MI**	***p*-value**
Age (years) mean ± SD	57.4 (0.9)	60.7 (0.8)	9.7E-24
BMI (Kg/m^2^)	26.6 (4.8)	27.2 (5.1)	5.2E-05
Waist-to-hip ratio	0.97 (0.08)	1 (0.08)	1.1E-10
Fat mass (%)	23.1 (8.7)	26.6 (10.4)	2.2e-16
Fasting plasma glucose (mmol/L)	5.8 (0.8)	5.9 (0.9)	2.6E-06
Fasting plasma insulin (mu/L)	6.9 (6.7)	7.8 (7.8)	1.2E-09
HbA1C (%)	5.7 (0.5)	5.9 (0.6)	2.2E-16
HOMA-IR	1.8 (1.9)	2.1 (2.3)	8.2E-11
Total cholesterol (mmol/L)	5.2 (1.3)	5.1 (1.3)	0.68
LDL cholesterol (mmol/L)	3.2 (1.2)	3.1 (1.2)	0.40
HDL cholesterol (mmol/L)	1.3 (0.5)	1.1 (0.5)	6.5E-10
Total triglyceride (mmol/L)	1.2 (0.8)	1.4 (0.7)	3.8E-07
Systolic blood pressure (mmHg)	136 (21.3)	142 (24)	1.0E-10
Diastolic blood pressure (mmHg)	87.3 (12.5)	87.6 (12.7)	0.32
GFR	0.09 (0.01)	0.08 (0.02)	2.2E-07
CRP (mg/L)	1.09 (1.8)	1.5 (2.6)	1.3E-07
Total alcohol/week (g)	61 (136)	61.5 (136.5)	4.7E-06
**Smoking [*****n*** **(%)]**			
No	3,946 (41)	204 (35)	1.6E-05
Yes	1,696 (18)	147 (25)	
Ex-smoker	3,965 (41)	239 (40)	
**Statins [*****n*** **(%)]**			
No	6,967 (73)	383 (65)	7.8E-05
Yes	2,640 (27)	207 (35)	

MI, myocardial infarction; SD, standard deviation; BMI, body mass index; HbA1c, hemoglobin A1c; HOMA-IR, homeostatic model assessment of insulin resistance; LDL, low-density lipoprotein; HDL, high-density lipoprotein; GFR, glomerular filtration rate; CRP, C-reactive protein.

**Figure 1:**
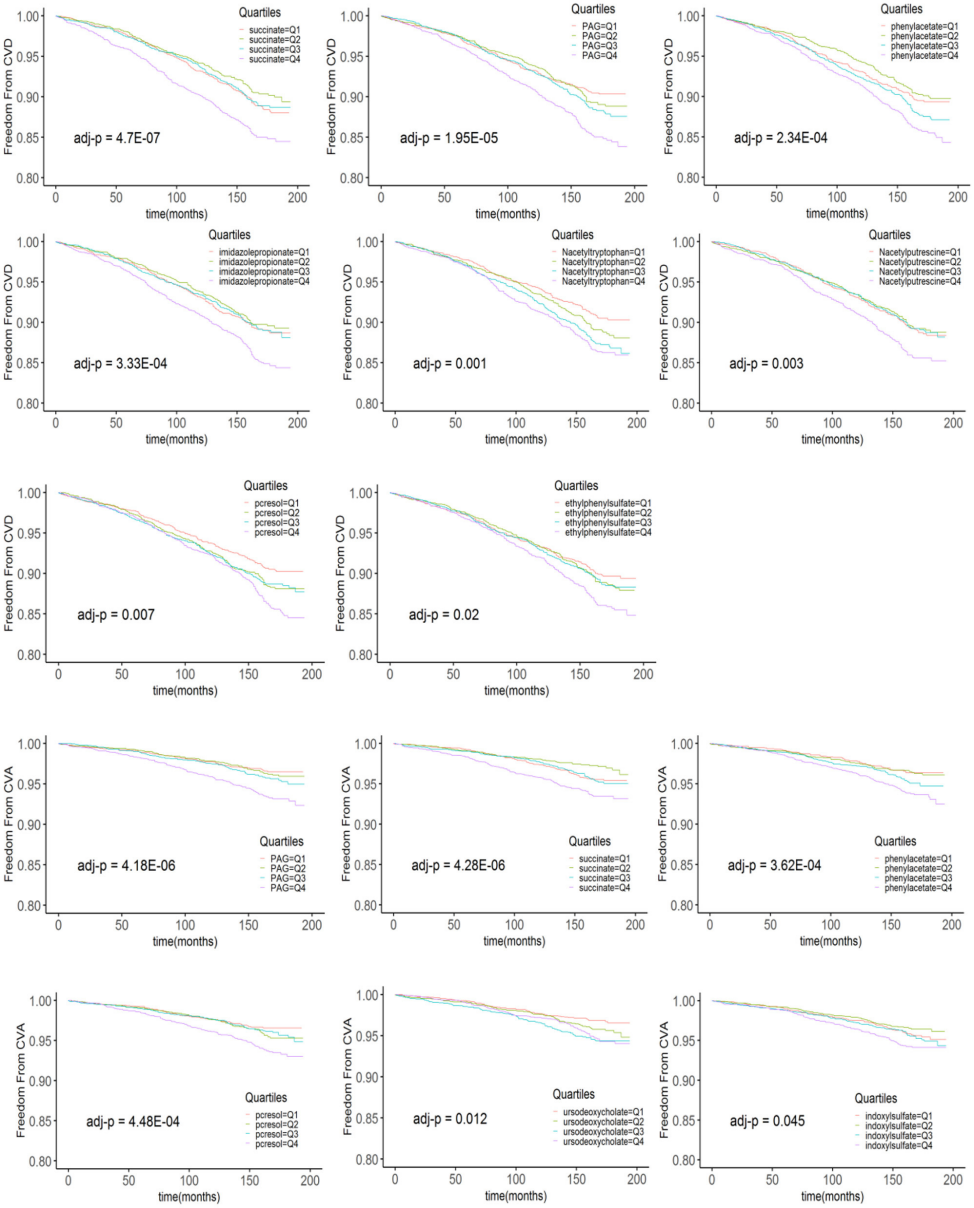
The Kaplan–Meier estimates and the risk of incident CVD and CVA over the follow-up period are stratified according to the quartiles of plasma metabolite levels. *p*-values derived from the log rank test are indicated and adjusted for multiple comparisons using the Bonferroni correction. PAG, phenylacetylglutamine.

The levels of metabolites at baseline in patients with and without CVD and CVA are shown in Table 3. After adjusting for multiple comparisons, no significant differences were found in the levels of metabolites between patients with MI and those without MI. In the Kaplan–Meier analysis, it was observed that patients in the highest quartile (Q4) of the metabolites had a significantly higher risk of CVD and CVA compared to patients in the lowest quartile. However, patients in the Q3 level of ursodeoxycholic acid (UDCA) had a higher risk of CVA compared to those in the Q1 level. In addition, the Kaplan–Meier curves of phenylacetylglutamine (PAG) and N-acetyltryptophan showed a dose-dependent increased risk of CVD (Figure 1). After adjusting for multiple comparisons, the Kaplan–Meier curves for metabolite concentrations in relation to MI events became non-significant.

**Table 3:** Comparison of gut microbe-derived metabolite concentrations between participants in the non-CVD and CVD groups, and between those in the non-CVA and CVA groups.

**Gut microbe-derived metabolites**	**Non-CVD Mean SD**	**CVD Mean SD**	**Adjusted *p*-value**
N-acetyltryptophan	−0.005 (0.35)	−0.05 (0.34)	4.6E−05
Phenylacetylglutamine (PAG)	−0.10 (0.70)	0.001 (0.69)	6.3E−05
p-cresol	−0.18 (90)	−0.07 (83)	0.0014
Phenylacetate	−0.17 (0.76)	−0.07 (0.79)	0.0031
Succinate	−0.11 (0.76)	−0.17 (0.88)	0.015
	**Non–CVA Mean SD**	**CVA Mean SD**	**Adjusted** ***p*****-value**
Phenylacetylglutamine (PAG)	−0.09	0.08	2.3E−05
Phenylacetate	−0.16	−0.0007	0.0015
Ursodeoxycholate	−0.09	0.07	0.035

CVD, cardiovascular disease; CVA, cerebrovascular accident; SD, standard deviation.

In the METSIM cohort, higher succinate levels (Q4. vs. Q1.) were associated with a 1.41-fold increased risk of CVD at the follow-up period of 200 months [unadjusted HR = 1.41; 95% CI, (1.18–1.7) adj. *p* < 0.001]. After adjusting for traditional CVD risk factors, higher levels of succinate were associated with a 1.3-fold increased risk of CVD [adjusted HR = 1.30; 95% CI, (1.10–1.53) *p* = 0.0003, adjusted *p* = 0.01]. We also examined the association between succinate and CVD as a continuous variable, which improved the robustness of the analysis (Bennette and Vickers, 2012). Interestingly, as a continuous variable, succinate was associated with a decreased risk of CVD [unadjusted HR = 0.91; 95% CI, (0.85–0.98) *p* = 0.016; adjusted HR = 0.9; 95% CI, (0.84–0.97) *p* = 0.008]. We then examined the association between succinate and metabolic disorders traits, including diabetes, obesity, and lipids, at baseline. After adjusting for traditional risk factors, significant inverse associations between succinate and plasma insulin levels (β = −0.02, *p* = 4.5E-04), homeostatic model assessment of insulin resistance (HOMA-IR; β = −0.023, *p* = 4.2E-04), BMI (β = −0.004, *p* = 0.002), and waist-to-hip ratio (β = −0.001, *p* = 6.0E-04) were observed, and a positive association was observed with HDL-C (β = 0.010, *p* = 4.4E-04).

**Figure 2:**
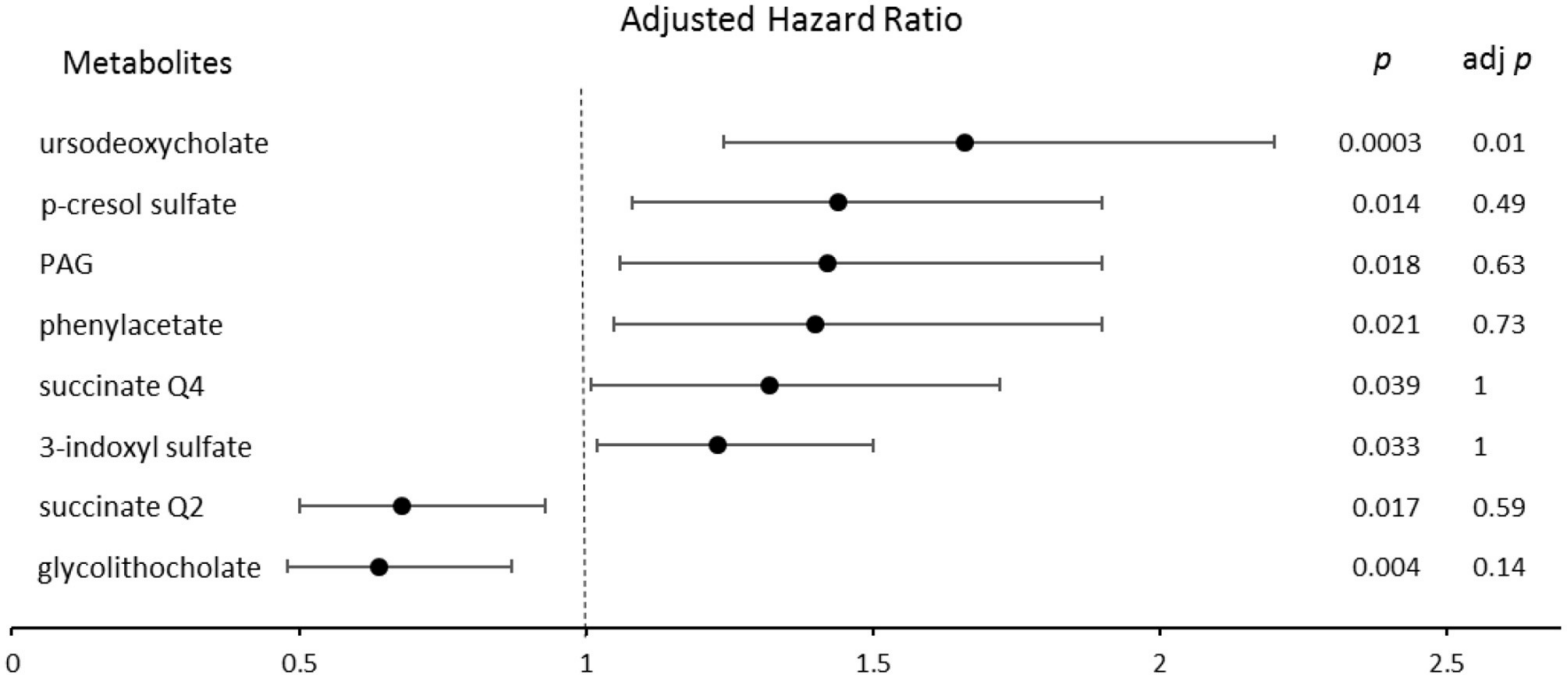
The forest plot indicating the risks of CVA incident 200 months following enrollment, stratified by the quartiles of gut microbe-derived metabolites levels. The multivariable Cox model for hazard ratios included adjustments for traditional risk factors. The 95% confidence interval is indicated by the length of the line. PAG, phenylacetylglutamine; Q, quartile.

A forest plot indicating the risk of incident CVA stratified by quartile gut microbe-derived metabolite levels after adjusting for traditional risk factors is shown in Figure 2. UDCA remained a significant predictor of CVA [unadjusted HR = 1.7; 95% CI, (1.3–2.3) *p* = 0.0002, adj. *p* = 0.007], even after adjusting for covariates and the Bonferroni correction [adjusted HR = 1.68; CI, (1.26–2.2) *p* = 0.0003, adj. *p* = 0.01]. Only succinate as a continuous variable remained a significant predictor for MI [unadjusted HR = 0.85; CI, (0.77–0.94) *p* = 0.0009, adj. *p* = 0.03], even after adjusting for traditional risk factors [adjusted HR = 0.85; CI, (0.77–0.93) *p* = 0.0007, adj. *p* = 0.024]. After adjusting for traditional risk factors, no association between PAG, CVD, and MI was found. A significant association between PAG [unadjusted HR = 1.5; CI, (1.3–1.7) *p* = 3.86e-07] and CVA [adjusted HR = 1.42; CI, (1.06–1.9) *p* = 0.018] was noted; however, after the correction for multiple comparisons, the *p*-value became non-significant. All 35 gut microbe-derived metabolites are shown in Table 4.

**Table 4:** Gut microbe-derived metabolites included in statistical analyses.

**Metabolites generated by microbiota**	
**Choline metabolism**	Phenylacetylglutamine (PAG)
Trimethylamine N-oxide (TMAO)	Phenol sulfate
**Bile acids**	Indolelactate
Glycocholate	Indoleacetate
Taurochenodeoxycholate	N-acetyltryptophan
Ursodeoxycholate	3-indoxyl sulfate
Glycodeoxycholate	Indoleacetylglutamine
Glycolithocholate sulfate	3-phenylpropionate (hydrocinnamate)
Glycocholenate sulfate	3-(3-hydroxyphenyl) propionate
Taurocholenate sulfate	**Non-aromatic amino acid metabolism**
Glycoursodeoxycholate	3-aminoisobutyrate
**Aromatic amino acid metabolism**	Imidazole propionate
P-cresol sulfate	**Lipid metabolism short-chain fatty acids**
Methyl indole-3-acetate	Isovalerate
Indolepropionate	Caproate
Phenyllactate	**Other metabolites**
Xanthurenate	Succinate
Phenylacetate	N-acetyl putrescine
3-(4-hydroxyphenyl) lactate	Spermidine
4-ethylphenylsulfate	Hippurate
4-hydroxyphenylacetate	

## 4 Discussion

The present study suggested that high plasma levels of succinate and UDCA are potential risk markers of CVD and CVA, respectively. In addition, succinate was found to be inversely associated with insulin resistance and obesity and positively associated with HDL-C.

The role of succinate continues to be a topic of discussion (Fernandez-Veledo and Vendrell, 2019). Succinate is an important metabolite of both host cell mitochondria and gut microbes. It is an intermediate product of the tricarboxylic acid cycle and serves as an inflammatory signal in the host. It has been shown to be a key mediator, via IL-1β, of macrophage response to lipopolysaccharide (Mills et al., 2016). In addition, gut microbes produce succinate as a byproduct of anaerobic fermentation. An increased abundance of succinate-producing bacteria is positively correlated with elevated plasma succinate levels and an increased risk of obesity (Serena et al., 2018). The concentrations of succinate are regulated by balancing succinate-consuming and succinate-producing bacterial strains. An imbalance in the succinate-producing and succinate-consuming bacterial strains in the gut results in the intestinal accumulation of succinate (Fernandez-Veledo and Vendrell, 2019). Succinate is typically detected at a relatively low concentration in the intestinal lumen, and it is rapidly converted into an intermediate in the production of propionate, although exact values can vary depending on the microbial species and the sample type (Wullt et al., 2007; Louis and Flint, 2017). The levels of succinate are reported at median concentrations of 15.3 μM in the plasma of healthy individuals (Kushnir et al., 2001) and have been found to increase up to 35.5 μM in patients with aortic aneurysm and dissection (Cui et al., 2021). The high plasma levels of succinate can activate SUCNR1 and signal transduction in macrophages and smooth muscle cells. SUCNR1 is inactive in normal tissues and can be activated under certain conditions, such as hypoxia and tissue injury (Fernandez-Veledo et al., 2021). During inflammatory responses, depending on cellular subsets, succinate plays crucial dual roles, which results in inflammation or anti-inflammation (Grimolizzi and Arranz, 2018). The binding of succinate to SUCNR1, which is expressed in human umbilical vein endothelial cells and macrophages, activates the transcription factor hypoxia-inducible factor (HIF)-1α, stimulates the succinate/IL-1β signaling axis, promotes the expression of IL-1β to produce excess pro-inflammatory cytokines, and exacerbates the inflammatory process of atherosclerosis (Tannahill et al., 2013; Xu et al., 2022). Elevated succinate levels in plasma were found to be associated with increased cardiovascular risk factors in young adults (Osuna-Prieto et al., 2021) and cardiac hypertrophy associated with obstructive coronary artery disease (Aguiar et al., 2014). Xu et al. observed that the arterial serum succinate level right before angiography was significantly elevated in patients with coronary heart disease compared to healthy controls (Xu et al., 2022). Another study demonstrated that the level of succinate in the coronary sinus was significantly elevated in patients with ST-elevation MI (Mills et al., 2016). At lower levels, succinate may offer metabolic and health benefits. A study conducted on animals suggested that *Prevotella copri* produces succinate that improved glucose homeostasis and body weight gain by serving as an intestinal gluconeogenic substrate. Increased cecal levels of succinate after the supplementation of fructo-oligosaccharides resulted in the activation of intestinal gluconeogenesis, which helped prevent obesity in a glucose-intolerant phenotype of mice fed a high-fat and high-sucrose diet (De Vadder et al., 2016).

In our analysis, UDCA, a secondary bile acid, was found to be a significant predictor of CVA. Patients with CVA had increased levels of UDCA in the serum compared to those in the non-CVA group. Manufactured UDCA has been used for decades to treat cholestatic liver diseases (de Vries and Beuers, 2017). UDCA has been shown to penetrate the cerebrospinal fluid in a dose-dependent manner (Parry et al., 2010). It has demonstrated neuroprotective effects in the rotenone model of Parkinson's disease by modulating the inflammatory apoptotic cascade (Abdelkader et al., 2016). Zhang et al. demonstrated that UDCA levels are lower in patients with CVA compared to those without CVA (Zhang et al., 2024). The authors measured the levels of UDCA after a stroke, and their sample size was relatively small (*n* = 56). In addition, in our previous study where we examined the associations between gut microbe-derived metabolites and metabolic syndrome traits in the METSIM cohort, we found significant associations between UDCA, insulin resistance, BMI, and total cholesterol (Mirzaei et al., 2024).

A significant elevation of PAG, a synthetic product of phenylacetate produced by the liver, was observed in cases of CVD and CVA. However, we did not find any significant association between PAG and CVD, CVA, and MI after adjusting for traditional risk factors. The non-significant result may be due to lack of power rather than lack of effect. Hazen et al. were the first to reveal a positive correlation between PAG and platelet functions and thrombosis (Zhu et al., 2023). They showed that PAG can promote CVD via adrenergic receptors (Nemet et al., 2020). Recent studies have demonstrated that plasma PAG levels at the time of admission were elevated in patients with an ischemic stroke and were associated with short-term adverse outcomes (Yu et al., 2021a,b).

In a prospective study of two cohorts of participants without the evidence of acute coronary syndrome with a follow-up period of 3 years, elevated plasma PAG and p-cresol sulfate (Q4 vs. Q1 quartile) were associated with major adverse cardiovascular events, defined as death, MI, and stroke. The researchers also showed that uremic toxins, including p-cresol sulfate and indoxyl sulfate, were associated with incident adverse cardiac event risks among subjects with primarily preserved kidney function (Nemet et al., 2023). Their results differed from those of our analyses, where adjusting for multiple comparisons impacted the significant associations between these metabolites and the risk of CVD.

The present study has several strengths and limitations. The METSIM cohort is a large randomly selected population-based cohort. We used a very conservative threshold for statistical significance by using the Bonferroni correction for multiple comparisons in our analyses. The homogeneity of the sample allowed for the control of numerous potential confounding variables. Our study consisted of only middle-aged and elderly Finnish men; therefore, our findings need to be replicated in more diverse populations. The findings are not generalizable to minority populations or women. Finally, our study was an association study, and we could not establish a causal relationship between gut microbe-derived metabolites and CVD.

## Data availability statement

Restrictions apply to the availability of data generated or analyzed during this study to preserve the confidentiality of the participants Requests to access these datasets should be directed at: markku.laakso@uef.fi.

## Ethics statement

The studies involving humans were approved by the Ethics Committee of the University of Eastern Finland and Kuopio University Hospital approved the METSIM study, and this study was conducted in accordance with the Declaration of Helsinki. All study participants gave written informed consent. The studies were conducted in accordance with the local legislation and institutional requirements. The participants provided their written informed consent to participate in this study.

## Author contributions

SM: Conceptualization, Formal analysis, Funding acquisition, Methodology, Software, Visualization, Writing – original draft. HD: Conceptualization, Methodology, Project administration, Supervision, Writing – review & editing. RC: Conceptualization, Formal analysis, Methodology, Writing – review & editing. AC: Writing – review & editing. LFS: Writing – review & editing. ML: Data curation, Writing – review & editing, Resources. AJL: Conceptualization, Methodology, Resources, Supervision, Writing – review & editing.

## References

[B1] Abdelkader N. F. Safar M. M. Salem H. A. (2016). Ursodeoxycholic acid ameliorates apoptotic cascade in the rotenone model of parkinson's disease: modulation of mitochondrial perturbations. Mol. Neurobiol. 53, 810–817. 10.1007/s12035-014-9043-8 25502462

[B2] Aguiar C. J. Rocha-Franco J. A. Sousa P. A. Santos A. K. Ladeira M. Rocha-Resende C.. (2014). Succinate causes pathological cardiomyocyte hypertrophy through GPR91 activation. Cell Commun. Signal. 12:78. 10.1186/s12964-014-0078-2 25539979 PMC4296677

[B3] Benjamin E. J. Muntner P. Alonso A. Bittencourt M. S. Callaway C. W. Carson A. P.. (2019). Heart disease and stroke statistics-2019 update: a report from the American Heart Association. Circulation 139, e56–e528. 10.1161/CIR.0000000000000659 30700139

[B4] Bennette C. Vickers A. (2012). Against quantiles: categorization of continuous variables in epidemiologic research, and its discontents. BMC Med. Res. Methodol. 12:21. 10.1186/1471-2288-12-21 22375553 PMC3353173

[B5] Bjorkegren J. L. M. Lusis A. J. (2022). Atherosclerosis: recent developments. Cell 185, 1630–1645. 10.1016/j.cell.2022.04.004 35504280 PMC9119695

[B6] Brown J. M. Hazen S. L. (2015). The gut microbial endocrine organ: bacterially derived signals driving cardiometabolic diseases. Annu. Rev. Med. 66, 343–359. 10.1146/annurev-med-060513-093205 25587655 PMC4456003

[B7] Bubier J. A. Chesler E. J. Weinstock G. M. (2021). Host genetic control of gut microbiome composition. Mamm. Genome. 32, 263–281. 10.1007/s00335-021-09884-2 34159422 PMC8295090

[B8] Candela M. Biagi E. Brigidi P. O'Toole P. W. De Vos W. M. (2014). Maintenance of a healthy trajectory of the intestinal microbiome during aging: a dietary approach. Mech. Ageing Dev. 136–137, 70–75. 10.1016/j.mad.2013.12.004 24373997

[B9] Cui H. Chen Y. Li K. Zhan R. Zhao M. Xu Y.. (2021). Untargeted metabolomics identifies succinate as a biomarker and therapeutic target in aortic aneurysm and dissection. Eur. Heart J. 42, 4373–4385. 10.1093/eurheartj/ehab605 34534287 PMC11506060

[B10] De Vadder F. Kovatcheva-Datchary P. Zitoun C. Duchampt A. Backhed F. Mithieux G.. (2016). Microbiota-produced succinate improves glucose homeostasis via intestinal gluconeogenesis. Cell Metab. 24, 151–157. 10.1016/j.cmet.2016.06.013 27411015

[B11] de Vries E. Beuers U. (2017). Management of cholestatic disease in 2017. Liver Int. 37(Suppl 1), 123–129. 10.1111/liv.13306 28052628

[B12] Drouin N. Kloots T. Schappler J. Rudaz S. Kohler I. Harms A.. (2019). Electromembrane extraction of highly polar compounds: analysis of cardiovascular biomarkers in plasma. Metabolites 10:4. 10.3390/metabo10010004 31861366 PMC7022788

[B13] Fan Y. Pedersen O. (2021). Gut microbiota in human metabolic health and disease. Nat. Rev. Microbiol. 19, 55–71. 10.1038/s41579-020-0433-9 32887946

[B14] Fernandez-Veledo S. Ceperuelo-Mallafre V. Vendrell J. (2021). Rethinking succinate: an unexpected hormone-like metabolite in energy homeostasis. Trends Endocrinol. Metab. 32, 680–692. 10.1016/j.tem.2021.06.003 34301438

[B15] Fernandez-Veledo S. Vendrell J. (2019). Gut microbiota-derived succinate: friend or foe in human metabolic diseases? Rev. Endocr. Metab. Disord. 20, 439–447. 10.1007/s11154-019-09513-z 31654259 PMC6938788

[B16] Grimolizzi F. Arranz L. (2018). Multiple faces of succinate beyond metabolism in blood. Haematologica 103, 1586–1592. 10.3324/haematol.2018.196097 29954939 PMC6165802

[B17] Kushnir M. M. Komaromy-Hiller G. Shushan B. Urry F. M. Roberts W. L. (2001). Analysis of dicarboxylic acids by tandem mass spectrometry. High-throughput quantitative measurement of methylmalonic acid in serum, plasma, and urine. Clin. Chem. 47, 1993–2002. 10.1093/clinchem/47.11.1993 11673368

[B18] Laakso M. Kuusisto J. Stancakova A. Kuulasmaa T. Pajukanta P. Lusis A. J.. (2017). The metabolic syndrome in men study: a resource for studies of metabolic and cardiovascular diseases. J. Lipid Res. 58, 481–93. 10.1194/jlr.O072629 28119442 PMC5335588

[B19] Louis P. Flint H. J. (2017). Formation of propionate and butyrate by the human colonic microbiota. Environ. Microbiol. 19, 29–41. 10.1111/1462-2920.13589 27928878

[B20] Mills E. L. Kelly B. Logan A. Costa A. S. H. Varma M. Bryant C. E.. (2016). Succinate dehydrogenase supports metabolic repurposing of mitochondria to drive inflammatory macrophages. Cell 167, 457–470.e13. 10.1016/j.cell.2016.08.064 27667687 PMC5863951

[B21] Mirzaei S. DeVon H. A. Cantor R. M. Cupido A. J. Pan C. Ha S. M.. (2024). Relationships and Mendelian randomization of gut microbe-derived metabolites with metabolic syndrome traits in the METSIM Cohort. Metabolites 14 :174. 10.3390/metabo14030174 38535334 PMC10972019

[B22] Nemet I. Li X. S. Haghikia A. Li L. Wilcox J. Romano K. A.. (2023). Atlas of gut microbe-derived products from aromatic amino acids and risk of cardiovascular morbidity and mortality. Eur. Heart J. 44, 3085–3096. 10.1093/eurheartj/ehad333 37342006 PMC10481777

[B23] Nemet I. Saha P. P. Gupta N. Zhu W. Romano K. A. Skye S. M.. (2020). A cardiovascular disease-linked gut microbial metabolite acts via adrenergic receptors. Cell. 180, 862–877.e22. 10.1016/j.cell.2020.02.016 32142679 PMC7402401

[B24] Osuna-Prieto F. J. Martinez-Tellez B. Ortiz-Alvarez L. Di X. Jurado-Fasoli L. Xu H.. (2021). Elevated plasma succinate levels are linked to higher cardiovascular disease risk factors in young adults. Cardiovasc. Diabetol. 20:151. 10.1186/s12933-021-01333-3 34315463 PMC8314524

[B25] Parry G. J. Rodrigues C. M. Aranha M. M. Hilbert S. J. Davey C. Kelkar P.. (2010). Safety, tolerability, and cerebrospinal fluid penetration of ursodeoxycholic acid in patients with amyotrophic lateral sclerosis. Clin. Neuropharmacol. 33, 17–21. 10.1097/WNF.0b013e3181c47569 19935406

[B26] Rehner J. Schmartz G. P. Kramer T. Keller V. Keller A. Becker S. L.. (2023). The effect of a planetary health diet on the human gut microbiome: a descriptive analysis. Nutrients 15:1924. 10.3390/nu15081924 37111144 PMC10144214

[B27] Roth G. A. Mensah G. A. Johnson C. O. Addolorato G. Ammirati E. Baddour L. M.. (2020). Global burden of cardiovascular diseases and risk factors, 1990-2019: update from the GBD 2019 study. J. Am. Coll. Cardiol. 76, 2982–3021. 10.1016/j.jacc.2020.11.010 33309175 PMC7755038

[B28] Serena C. Ceperuelo-Mallafre V. Keiran N. Queipo-Ortuno M. I. Bernal R. Gomez-Huelgas R.. (2018). Elevated circulating levels of succinate in human obesity are linked to specific gut microbiota. ISME J. 12, 1642–1657. 10.1038/s41396-018-0068-2 29434314 PMC6018807

[B29] Spanogiannopoulos P. Bess E. N. Carmody R. N. Turnbaugh P. J. (2016). The microbial pharmacists within us: a metagenomic view of xenobiotic metabolism. Nat. Rev. Microbiol. 14, 273–287. 10.1038/nrmicro.2016.17 26972811 PMC5243131

[B30] Stancakova A. Javorsky M. Kuulasmaa T. Haffner S. M. Kuusisto J. Laakso M.. (2009). Changes in insulin sensitivity and insulin release in relation to glycemia and glucose tolerance in 6,414 Finnish men. Diabetes. 58, 1212–1221. 10.2337/db08-1607 19223598 PMC2671053

[B31] Tannahill G. M. Curtis A. M. Adamik J. Palsson-McDermott E. M. McGettrick A. F. Goel G.. (2013). Succinate is an inflammatory signal that induces IL-1beta through HIF-1alpha. Nature 496, 238–242. 10.1038/nature11986 23535595 PMC4031686

[B32] Vangipurapu J. Stancakova A. Smith U. Kuusisto J. Laakso M. (2019). Nine amino acids are associated with decreased insulin secretion and elevated glucose levels in a 7.4-year follow-up study of 5,181 Finnish men. Diabetes 68, 1353–1358. 10.2337/db18-1076 30885989

[B33] Witkowski M. Weeks T. L. Hazen S. L. (2020). Gut microbiota and cardiovascular disease. Circ. Res. 127, 553–570. 10.1161/CIRCRESAHA.120.316242 32762536 PMC7416843

[B34] Wullt M. Johansson Hagslatt M. L. Odenholt I. Berggren A. (2007). *Lactobacillus plantarum* 299v enhances the concentrations of fecal short-chain fatty acids in patients with recurrent clostridium difficile-associated diarrhea. Dig. Dis. Sci. 52, 2082–2086. 10.1007/s10620-006-9123-3 17420953

[B35] Xu J. Zheng Y. Zhao Y. Zhang Y. Li H. Zhang A.. (2022). Succinate/IL-1beta signaling axis promotes the inflammatory progression of endothelial and exacerbates atherosclerosis. Front. Immunol. 13:817572. 10.3389/fimmu.2022.817572 35273600 PMC8901997

[B36] Yin X. Chan L. S. Bose D. Jackson A. U. VandeHaar P. Locke A. E.. (2022). Genome-wide association studies of metabolites in Finnish men identify disease-relevant loci. Nat. Commun. 13:1644. 10.1038/s41467-022-29143-5 35347128 PMC8960770

[B37] Yu F. Feng X. Li X. Luo Y. Wei M. Zhao T.. (2021a). Gut-derived metabolite phenylacetylglutamine and white matter hyperintensities in patients with acute ischemic stroke. Front. Aging Neurosci. 13:675158. 10.3389/fnagi.2021.675158 34393756 PMC8363199

[B38] Yu F. Li X. Feng X. Wei M. Luo Y. Zhao T.. (2021b). Phenylacetylglutamine, a novel biomarker in acute ischemic stroke. Front. Cardiovasc. Med. 8:798765. 10.3389/fcvm.2021.798765 35004911 PMC8733610

[B39] Zhang F. Deng Y. Wang H. Fu J. Wu G. Duan Z.. (2024). Gut microbiota-mediated ursodeoxycholic acids regulate the inflammation of microglia through TGR5 signaling after MCAO. Brain Behav. Immun. 115, 667–679. 10.1016/j.bbi.2023.11.021 37989444

[B40] Zhu Y. Dwidar M. Nemet I. Buffa J. A. Sangwan N. Li X. S.. (2023). Two distinct gut microbial pathways contribute to meta-organismal production of phenylacetylglutamine with links to cardiovascular disease. Cell Host Microbe. 31, 18–32.e9. 10.1016/j.chom.2022.11.015 36549300 PMC9839529

